# Multi-site validation of an interpretable model to analyze breast masses

**DOI:** 10.1371/journal.pone.0320091

**Published:** 2025-06-26

**Authors:** Luke Moffett, Alina Jade Barnett, Jon Donnelly, Fides Regina Schwartz, Hari Trivedi, Joseph Lo, Cynthia Rudin

**Affiliations:** 1 Department of Computer Science, Duke University, Durham, North Carolina, United States of America; 2 Department of Radiology, Brigham and Women’s Hospital, Boston, Massachusetts, United States of America; 3 Department of Radiology and Imaging Services, Emory University, Atlanta, Georgia, United States of America; Medical University of Vienna, AUSTRIA

## Abstract

An external validation of IAIA-BL—a deep-learning based, inherently interpretable breast lesion malignancy prediction model—was performed on two patient populations: 207 women ages 31 to 96, (425 mammograms) from iCAD, and 58 women (104 mammograms) from Emory University. This is the first external validation of an inherently interpretable, deep learning-based lesion classification model. IAIA-BL and black-box baseline models had lower mass margin classification performance on the external datasets than the internal dataset as measured by AUC. These losses correlated with a smaller reduction in malignancy classification performance, though AUC 95% confidence intervals overlapped for all sites. However, interpretability, as measured by model activation on relevant portions of the lesion, was maintained across all populations. Together, these results show that model interpretability can generalize even when performance does not.

## Introduction

Many leading radiology organizations have cited *transparency and explainability* as one of the key ethical concerns for the adoption of artificial intelligence in radiology [1]. In mammography, few models achieve this goal—in fact, a 2023 literature review by Loizidou *et al*. covering computer aided detection (CAD) papers for mammography found only one out of 90 models proposed in the papers was inherently interpretable [2]. That model is IAIA-BL, an interpretable AI algorithm for breast lesions, proposed by Barnett *et al*. in 2021 [3,4]. The model showed promising results both for predicting lesions and for providing interpretable explanations of its reasoning, but the tests were performed on a private, in-house Duke University dataset that did not include external validation. In this paper, we address this by presenting a multi-site external validation of IAIA-BL using data from Emory University and the CAD vendor iCAD, studying not only the accuracy of the model but also the ability of its interpretable reasoning process to generalize.

IAIA-BL is a breast lesion classifier that makes two separate classifications using images of breast lesions. The model is provided a region of interest (ROI) extracted from a mammogram that is centered on a lesion. It first classifies the lesion margin using the standard Breast Imaging Reporting and Database System (BI-RADS) mass margin categorization [5]. It then combines the probabilities assigned to each margin type to predict whether or not the lesion is malignant.

IAIA-BL relies on learning and predicting mass margins of masses in regions of interest. Mass margin labels are not available in most common public breast imaging datasets even though those margins are a standard part of the BI-RADS lexicon. On the paper’s in-house test set of 78 images from 49 patients, IAIA-BL achieved an area under the receiver operator characteristic curve (AUC) of 0.951 (95% CI: 0.905, 0.996) for predicting mass margins on three margin classes: circumscribed, indistinct, and spiculated. It achieved an AUC of 0.84 (95% CI: 0.74, 0.94) for malignancy prediction on the same dataset. There are no other public lesion or malignancy models that have reported performance on this data set for comparison, though the authors found that IAIA-BL achieved performance on-par with standard (i.e., black box) vision model baselines.

The first step in IAIA-BL’s prediction chain, classifying the margins, uses the “this looks like that” style of reasoning first introduced by Chen *et al*. [6] and Li *et al*., [7]. To do so, IAIA-BL compares the margins of the lesion being evaluated to a set of prototypical lesions learned in training. The final margin classification is determined by the margins of the prototype images *most similar* to the lesion under evaluation. This kind of reasoning is intended to be interpretable: a radiologist can “fact check” IAIA-BL’s reasoning by evaluating the similarity between the lesion and the prototypes.

IAIA-BL’s primary innovation is a training method that allows the use of expert pixel-level lesion annotations to guide the model’s learning. Using these annotations and a *fine annotation loss*, IAIA-BL learns to separate the lesion’s margin from the surrounding background tissue and to use only the lesion and its margin for its predictions (see [3] for a formal definition of fine annotation loss).

IAIA-BL then uses a logistic regression over the raw margin class score for all margin types to predict malignancy. This is also interpretable because each margin type has a single learned malignancy weight. For instance, circumscribed margins, which have a low likelihood of malignancy, have a learned negative malignancy weight. Conversely, spiculated margins, which are commonly associated with invasive cancers, have a high positive malignancy weight.

**Fig 1:**
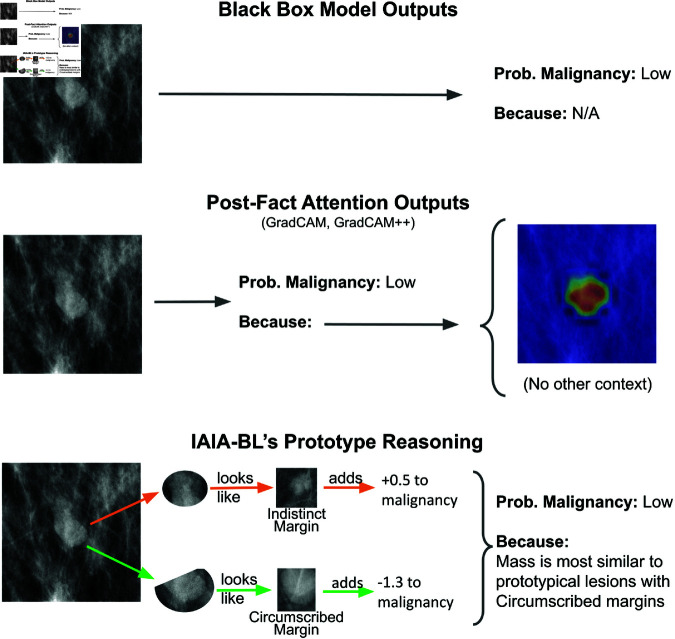
Comparing reasoning of IAIA-BL to alternative models. *Top: Black-Box Models.* Black-box models provide only a final prediction. In our study, we use VGG16 as a representative black box model. *Middle: Activation Maps.* Activation mapping introspects the neutral network to find which pixels lead to its prediction, but does not provide an explanation of why those pixels are relevant. In our study, we apply GradCAM and GradCAM++ to VGG16 as a representative activation mapping approach. In this case, the method simply highlighted the lesion and provided no useful information. *Bottom: IAIA-BL.* IAIA-BL makes two predictions—mass margin and malignancy—broken into two phases: First, it inspects the region of interest to find prototypical lesions that look like the lesion in the region of interest. It uses the margin labels from those prototypical lesions to assign a score to each margin class. Second, it uses the weighted scores for each margin label to predict malignancy. Circumscribed margins lower the likelihood of malignancy, whereas indistinct margins increase the malignancy likelihood moderately and spiculated margins significantly. This figure is adapted from Barnett *et al*. [9].

Fig 1 compares IAIA-BL’s reasoning process to approaches that are not inherently interpretable. In the example provided, the lesion margin shows similarity to prototypical indistinct and circumscribed lesions. The reasoning process is transparent, showing not only the margin predictions and scores, but also the prototypes from which the model learned the margins. This reasoning process also replicates one task radiologists undertake when reading mammograms, namely lesion margin classification using BI-RADS. The model architecture for IAIA-BL is described in detail by Barnett *et al*. [3].

To execute the external validation, datasets were collected from Emory University and the vendor iCAD. From Emory University, all images are part of the EMBED dataset [8]. In addition to the complete EMBED dataset, subset results are reported on the publicly available EMBED Open Data (see S1 Appendix), https://registry.opendata.aws/emory-breast-imaging-dataset-embed/. The iCAD dataset is a proprietary dataset used for model training and evaluation. Cases were selected from both datasets based on the availability of ROIs, BI-RADS margin labels, and confirmed histopathology. Using the model originally trained by Barnett *et al*. [4], the evaluations were repeated for both external datasets, along with evaluations of baseline models trained on the **Competing interest:** LM: no completing interests to declare. AJB: no competing interests to declare. JD: no competing interests to declare. HT: no competing interests to declare. FS: no competing interests to declare. JL: I have read the journal’s policy and the authors of this manuscript have the following competing interests: royalties/license from iCAD. CR: no competing interests to declare. This does not alter our adherence to PLOS ONE policies on sharing data and materials. same dataset as IAIA-BL.

We find that none of the models we test perform as well on the external datasets, averaging a 0.12 AUC drop in malignancy prediction performance. We attribute this model-independent drop in performance to shifts in data distribution (as well as the small size of datasets available for mammography studies). However, we also find that, regardless of site and unlike the baseline models, IAIA-BL’s lesion-only interpretable reasoning process does generalize across sites. We conclude with a discussion of the methodological implications of these seemingly incompatible findings.

## Related work

Computer aided breast cancer classification has long been an objective of computer science and radiology research [2]. Recent work on this goal has focused on the application of deep learning methods to breast cancer classification, with promising results—several studies [10–13] reported predictive accuracies of at least 97% on their respective test datasets. However, each of these methods produces inscrutable predictions because they use black box models for prediction. Post-hoc methods for explaining such models exist [14,15], but such explanations are not generally faithful to the actual reason the network made a prediction [16–18]. IAIA-BL [3] is an interpretable alternative to such models, following a simple, case-based approach to breast cancer classification.

## Materials and methods

This retrospective study was HIPAA-compliant and approved by the Duke Health IRB. Informed consent for this retrospective study was waived by the Duke Health IRB.

### Evaluation criteria

IAIA-BL’s original evaluation measured performance using three metrics that are replicated in this external validation. First, the model was assessed on its ability to correctly classify BI-RADS mass margins as measured by one-versus-all AUC for three margin classes: circumscribed, indistinct, and spiculated. iCAD labels only indicate whether the margin is spiculated, so other classes (i.e., circumscribed, indistinct) are omitted for iCAD.

Second, IAIA-BL was assessed on the ability to predict malignancy from the learned malignancy weight it placed on each margin classification as measured by AUC. 95% confidence intervals for both types of AUC were calculated using DeLong’s method. We also analyzed the distribution of malignancy scores and produced confusion matrices for each model at each site. Confusion matrices were produced by choosing the highest operating point that achieved a less than 20% false positive rate on the Duke dataset; the same operating point was used for all sites.

**Fig 2:**
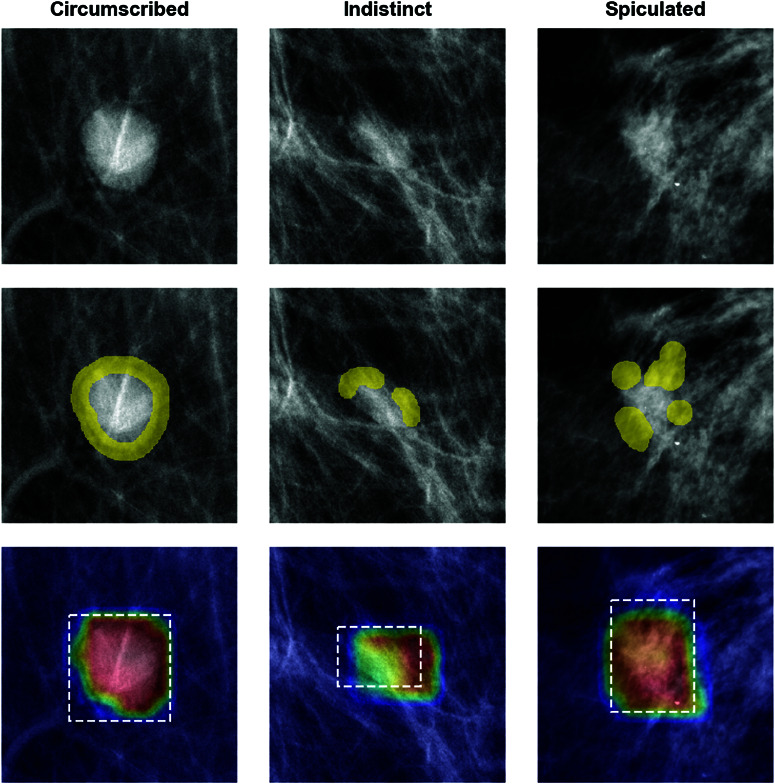
Fine annotations and activation precision. *Top Row:* Images of lesions from the Duke training dataset as they passed into IAIA-BL. *Middle Row:* Fine annotations on the lesion margins as provided by radiologist annotators. Fine annotations were introduced as a regularization to ProtoPNet-style models by Barnett *et al*. for IAIA-BL [4]. These fine annotations are provided to the model during training. They help the model learn the relevant part of the lesion (i.e., the margin) and not (potentially confounding) background information. *Bottom Row:* Activation map and region of interest (ROI) bounding box. ROI bounding boxes were created from lesion annotations for all the datasets. The bounding boxes in these images were created by bounding the fine annotations (see the Middle Row). The activation map shows the areas of the image that the model uses in its reasoning. These activation maps are from IAIA-BL, which are constructed from prototype similarity to patches of the image and therefore faithfully reflect the model reasoning. Activation precision is calculated as the proportion of high model activation contained within the ROI bounding box. In the circumscribed and spiculated examples, the activation precision is close to 1.0. In the indistinct example, the activation map protrudes beyond the bounding box, and the activation precision is closer to 0.5.

Third, a metric called *activation precision* measured the proportion of the model’s activations that come from information within the radiologist-provided regions of interest (ROIs), producing a value between 0.0 and 1.0. Activation precision measures how much of the model’s high activation is within the ROI, as measured by an activation map. The most common activation mapping technique is GradCAM [14], but this post-hoc method is not necessarily faithful to the model’s reasoning process [18]. In contrast, when performing inference using IAIA-BL, similarity between the learned prototypical parts of the model and parts of the image sample is used to construct the activation map, which is faithful to the model’s reasoning. These similarity scores are calculated for a grid of subsections embedded from the sample image. When calculating activation precision, this grid of similarities is upsampled to match the original image dimension. The model has high activation precision (close to 1.0) if the areas of high similarity are contained within the bounding box of the ROI—meaning that the model has determined that its learned prototypical lesions are similar to the lesion in the ROI as opposed to the background tissue. Activation precision is formally defined by Barnett *et al*. [4]. Examples of outputs from IAIA-BL showing model activation as a heatmap are in Fig 2. 95% confidence intervals for activation precision were calculated using bootstrap resampling (*n* = 100). All metrics and qualitative analyses were collected from all three sites and are presented in Results.

### External dataset preparation

The Emory University test set contained 103 images of 58 biopsied masses from 58 patients. A validation set of 100 images from 100 patients for context window tuning was also prepared, described in detail below. All images were from the dataset EMBED, described by Jeong *et al*. [8]. From iCAD, 425 images of 449 biopsied masses from 207 patients were included. The original dataset from Duke University had 1,136 images from 484 patients for all mass margins. Restricted to circumscribed, indistinct, and spiculated lesions, 375 images were used for training and 78 for testing. For both external datasets, images were annotated by their respective organizations; labels were taken from the metadata. IAIA-BL does not perform ROI extraction, so only images with indicated ROIs were included in the evaluation.

For this study, the Duke dataset was first accessed in September, 2022, the Emory dataset in February, 2023, and the iCAD dataset in April, 2023. All data used in this study was anonymized before it was accessed. Hari Trevedi also had access to the pre-anonymized data at Emory University, but the unanonymized data was not accessed as a part of this study.

For both sites, images were included in test sets if (1) the image was from a screening mammogram (restricted to standard screening mammogram views, craniocaudal (CC) and mediolateral oblique (MLO)); (2) the image was a full-field 2D image; (3) a region of interest’s bounding box was provided, indicating the location of the mass in the image; (4) the mass’ histopathology was confirmed through biopsy after the image was taken; (5) the mass had a label for the BI-RADS mass margin.

For EMBED, images were further restricted to the set with a single ROI per image; the data structure of EMBED does not allow mapping individual ROIs to histopathologies or margin labels when there are multiple ROIs in the same image. Margin labels were also restricted to circumscribed, indistinct, and spiculated, the margins IAIA-BL predicts.

For iCAD, margins were labeled either *spiculated* or *non-spiculated*. ROI annotations for iCAD were provided as paths tracing the edge of the lesion. These paths are provided almost exclusively for cases with positive histopathology as the iCAD dataset was prepared for cancer detection. IAIA-BL was provided ROIs constructed from the bounding box of these paths with a 100 pixel margin on each side.

**Fig 3:**
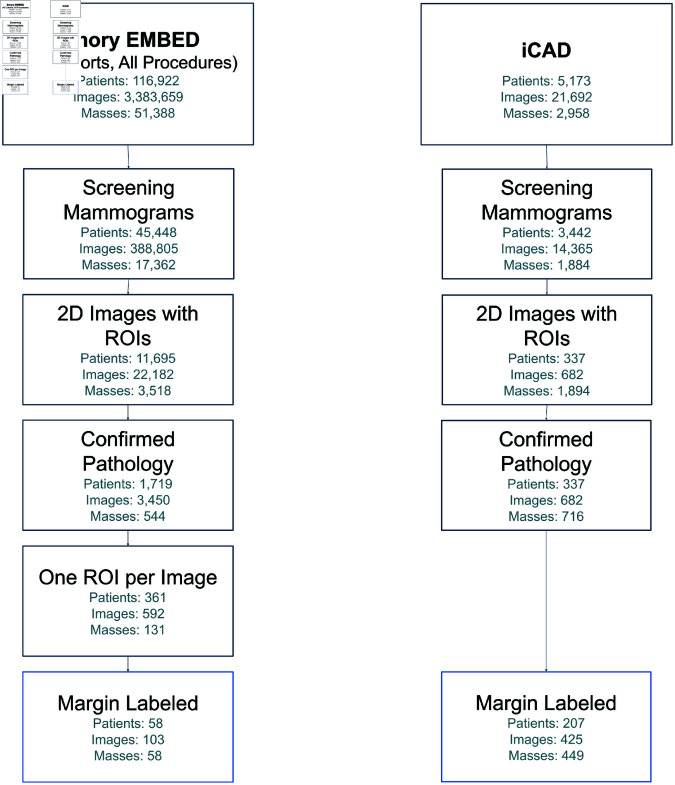
Selection flow chart for test sets. ROI (Region of Interest), an annotated bounding box around an identified lesion. Screen images are from screening exams and of the CC (craniocaudal) or MLO (mediolateral oblique) view. The selection process finds images that meet the four criteria. For Emory, mass margin labels are taken from either the original screening exam or a follow-up diagnostic exam within 6 months, with the diagnostic exam preferred. In cases where there are more than one ROI on the same image, the dataset does not allow attribution of histopathology and margin label to a single ROI, so those cases are omitted. For iCAD, margins are only labeled *spiculated* or *not spiculated*. Both margins are included in the test set for malignancy, but only performance on spiculated margins is reported for margin classification.

This resulted in the following distribution of margin labels for masses; from Emory University, 47 circumscribed, 39 indistinct, and 17 spiculated masses; from ICAD, 280 spiculated, and 169 not spiculated masses. In the original Duke test dataset, patient races were 56% Caucasian/White (44), 41% Black or African American (32), and 3% Other/Unknown (2). The Emory test set had a similar distribution, with 48% Black or African American (49), 45% Caucasian/White (47), and 7% Other/Unknown. The iCAD dataset did not include race demographics. Racial, age, and equipment manufacturer subgroups are reported in Table 1, mass margin distributions in Table 2, and the selection procedure in Fig 3.

**Table 1:** Patient test set demographics and pathologies.

	Duke	Emory	iCAD
Unique Patients	49	58	207
Unique Masses	77	58	449
Unique Images	78	103	425
*Manufacturer*			
GE	63	–	234
Hologic	15	103	124
Siemens	–	–	91
*Patient Age*			
≤50	13	31	47
50-69	43	47	263
≥70	22	25	139
*Race*			
Black or African American	32	49	–
Caucasian/White	44	47	–
Other/Unknown	2	7	207

All values represent the number of images except those in the Unique Patients and Unique Masses. “Other” race category includes Asian, Native Hawaiian or Other Pacific Islander, American Indian or Alaskan Native, and Unknown. Race demographics were not available for the iCAD data.

**Table 2:** Patient test set mass margins.

	Duke	Emory	iCAD
Circumscribed	26	47	–
Indistinct	33	39	–
Spiculated	33	17	280
Non-Spiculated	–	–	169

Distribution of mass margins in each test dataset. iCAD only labeled the masses as spiculated or non-spiculated.

Images that met the selection criteria were preprocessed by (1) extracting the images from DICOM files (this was completed on EMBED before the data was accessed for the study), (2) applying windowing and leveling to normalize the image histograms to be in [0,1], and (3) extracting ROIs into separate image files to provide to the model. Images from EMBED were renormalized to be in [0,1] after the application of the proprietary preprocessing.

A separate validation set was constructed from Emory data using the same criteria as the test set, except the margin labels were omitted from the criteria. The validation set was used on EMBED to tune the context window size. The *context window* is the set of extra pixels outside the ROI included in the image during preprocessing. Having a context window is necessary for calculating activation precision, but it also improved performance in the original paper. A random sample of 100 patients for Emory University meeting criteria 1–4 but not criteria 5 (lacking margin labels) was used as a validation set for tuning context window size. The final context window was 100 pixels added to each side of the bounding box (up to the edge of the image). These are the same values used in the original IAIA-BL paper on the Duke dataset. This context window was also used in preparing the iCAD dataset. A full description of the tuning process is available in S1 Appendix.

### Baseline models

Aside from IAIA-BL, two other deep-learning models trained on the Duke in-house dataset were also tested as baselines: ProtoPNet and VGG-16 (pre-trained on ImageNet) [19]. ProtoPNet is the general purpose ancestor of IAIA-BL and also inherently interpretable. Comparing against ProtoPNet allows us to measure effectiveness of IAIA-BL’s fine annotations (and other algorithmic improvements) in guiding the model to learn from the masses and their margins as measured by activation precision. VGG-16 is a general-purpose black-box vision model, which provides a baseline for the difficulty of margin prediction and malignancy prediction. Since VGG-16 is not inherently interpretable, GradCAM and GradCAM++ were used to measure activation precision [14,15]. Both models were also used as baselines in the original IAIA-BL paper. All three use a VGG-16 backbone pretrained on ImageNet [20], allowing us to compare the relative effects of the prototype reasoning and IAIA-BL’s medical imaging enhancements without confounding effects of different feature extractions. We re-finetuned VGG-16 for this paper to address a bug in the implementation used by Barnett *et al*. On the Duke dataset, this yielded a mean mass margin classification AUC of 0.92 (95% CI: 0.86, 0.98). All reported results use this corrected model.

### Training dataset

Complete details of model training are described in Barnett *et al*. [4], which we summarize here. A training set of 516 lesion images was collected from the Duke University Hospital system (125 spiculated, 220 indistinct, 171 circumscribed). Mass margin labels and ROIs were provided by a single fellowship trained radiologist, including 15 lesions with pixel-level fine annotations (five for each class). Data augmentation was performed using random flips, random rotations, and random cropping (up to 80% of the original lesion) such that the final training set contained 5,000 images. All models (IAIA-BL and baselines) were trained on this training set.

## Results

### Mass margin prediction

**Fig 4:**
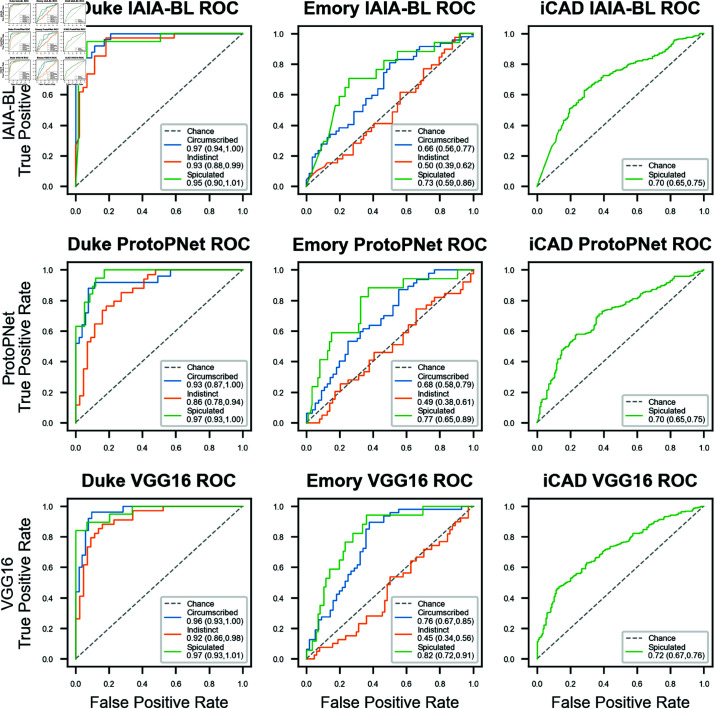
Margin classification ROC curves. ROC curves are provided for IAIA-BL, ProtoPNet, and the black box baseline VGG16. AUCs are reported in the legend, and 95% confidence intervals, calculated using DeLong’s method, are in parentheses. On iCAD, only spiculated mass classification is reported since other margin types are unknown.

Margin prediction AUC for all models are in Table 3 and ROC curves are Fig 4, which show all models experience similar mean margin prediction performance drops at each site.

**Table 3:** Margin prediction performance and activation precision.

			Duke	Emory	iCAD
Margin AUC	IAIA-BL		0.95 (0.91, 1.00)	0.63 (0.51, 0.75)	0.70 (0.65, 0.75)
	ProtoPNet		0.92 (0.86, 0.98)	0.65 (0.53, 0.76)	0.70 (0.65, 0.75)
	VGG16		0.95 (0.90, 1.00)	0.68 (0.57, 0.78)	0.72 (0.67, 0.76)
Activation Precision	IAIA-BL		0.94 (0.94, 0.95)	1.00 (0.99, 1.00)	0.92 (0.92, 0.92)
	ProtoPNet		0.56 (0.34, 0.79)	0.77 (0.53, 1.02)	0.55 (0.15, 0.95)
	VGG16	GradCAM	0.64 (0.53, 0.73)	0.72 (0.62, 0.81)	0.16 (0.14, 0.18)
		GradCAM++	0.70 (0.60, 0.80)	0.83 (0.73, 0.91)	0.43 (0.40, 0.47)

AUC measures one-versus-all classification performance for each margin; numbers reported are the averages across all three margin classes. The 95% confidence intervals are in parentheses, calculated using DeLong’s method for AUC and bootstrap resampling for activation precision (n = 100). VGG-16 does not have a self-explanation of activation, so GradCAM and GradCAM++ were used. All models experience similar decreases in performance in margin prediction when moving from the Duke in-house dataset to external datasets. On iCAD, only spiculated mass activation precision is reported since other margin types are unknown. Only IAIA-BL has consistently excellent activation precision (>.9) on all datasets, indicating it is not confounded by background noise.

On Emory’s EMBED, IAIA-BL achieved an average mass margin classification AUC for all classes of 0.63 (95% CI: 0.51, 0.75). In one-versus-all classification, it achieved 0.66 AUC (95% CI: 0.56, 0.77) for circumscribed margins, 0.50 AUC (95% CI: 0.39, 0.62) for indistinct margins, and 0.73 AUC (95% CI: 0.59, 0.86) for spiculated margins.

On iCAD, with only spiculated margins labeled, IAIA-BL achieved a spiculated margin classification AUC of 0.70 (95% CI: 0.65, 0.75), compared to base ProtoPNet (0.70, 95% CI: 0.65, 0.75) and VGG-16 (0.72, 95% CI: 0.67, 0.76).

### Activation precision

**Fig 5:**
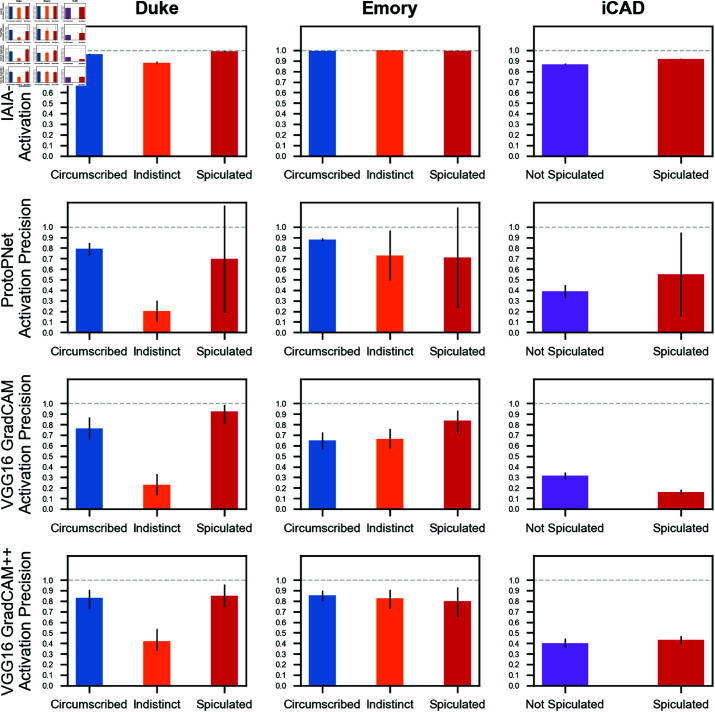
Margin level activation precision. Activation Precision of margin prediction for IAIA-BL and baseline models. VGG-16 does not have a self-explanation of activation, so GradCAM and GradCAM++ were used. Error bars represent 95% confidence intervals, calculated using bootstrap resampling (n=100). On iCAD, only spiculated mass activation precision is reported since other margin types are unknown. The differences in performance across margin type is stark, with indistinct margins performing poorly for both ProtoPNet and VGG16. The performance on the tightly drawn spiculated margins from iCAD shows differentiation across the models.

For activation precision, IAIA-BL achieved 1.00 (95% CI: 0.99, 1.00) and 0.92 (95% CI: 0.92, 0.92) at Emory and iCAD, respectively. This is compared to GradCAM++ on VGG16’s 0.83 (95% CI: 0.73, 0.91) and 0.43 (95% CI: 0.40, 0.47), and compared to ProtoPNet’s 0.77 (95% CI: 0.53, 1.02) and 0.55 (95% CI: 0.15, 0.95). Overall activation precisions are in Table 3 and margin level results are in Fig 5.

### Malignancy prediction

**Fig 6:**
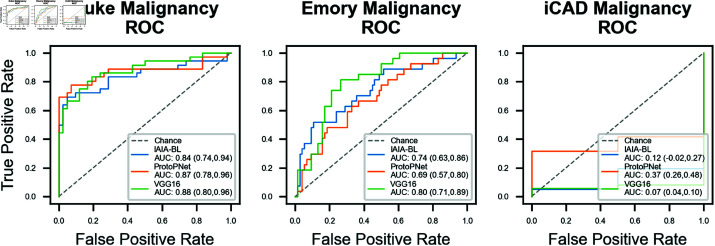
Malignancy prediction ROC curves. AUCs are reported in the legend. 95% confidence intervals, calculated using DeLong’s method, are in parentheses. All models experience an AUC drop from Duke to Emory, though the confidence intervals overlap in each case. iCAD only has 2 negative cases out of 449 cases, both of which have spiculated margins, which results in very low AUC values. The iCAD AUCs are reported for completeness.

**Fig 7:**
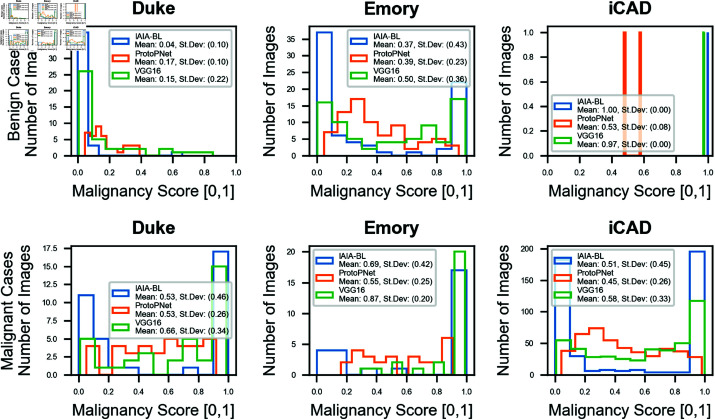
Malignancy prediction distributions. *Top Row:* Distribution of malignancy prediction scores by model and site on benign cases. *Bottom Row:* Distribution of malignancy prediction scores by model and site on malignant cases. Means and standard deviations are in parentheses in the legend. iCAD has only 2 negative cases. VGG-16 and IAIA-BL have near-1.0 scores for a large number of positive cases and near-0.0 scores for a large number of negative cases, whereas ProtoPNet’s scores are more evenly distributed.

On Emory University (EMBED), IAIA-BL achieved a malignancy prediction AUC of 0.84 (95% CI: 0.74, 0.94). Since only two cases in iCAD were not malignant (0.04%), AUC provides little information, though it is reported in Fig 6 for completeness. Alternatively, confusion matrices at the 20% false positive rate operating point are in Table 4, ROC curves for all models are in Fig 6, and distributions of malignancy scores are in Fig 7.

**Table 4:** Confusion matrices for malignancy prediction.

		Duke		Emory		iCAD	
		Pr. B.	Pr. M.	Pr. B.	Pr. M.	Pr. B.	Pr. M.
IAIA-BL	Actual Benign	24	18	11	65	0	2
	Actual Malignant	6	30	1	26	49	398
ProtoPNet	Actual Benign	34	8	35	41	0	2
	Actual Malignant	7	29	5	22	152	295
VGG16	Actual Benign	35	7	33	43	0	2
	Actual Malignant	7	29	1	26	135	312

*Pr. B.* is Predicted Benign and *Pr. M.* is Predicted Malignant. Operating points for the confusion matrices were chosen by finding the highest operating point that elicited a less than 20% false negative rate on the original Duke University dataset for each model. Those values are 0.03 for IAIA-BL, 0.29 for ProtoPNet, and 0.34 for VGG-16. At these operating points, IAIA-BL is less specific on Duke and Emory, but correctly identifies more cancers in iCAD’s 99.6% malignant dataset.

All of the models tested (IAIA-BL, ProtoPNet, or VGG-16) experienced a mean AUC drop when tested on the Emory dataset. For malignancy prediction on Emory data, it was 0.84 to 0.74 (−0.10) for IAIA-BL, 0.88 to 0.69 (−0.19) for ProtoPNet, and 0.88 to 0.80 (−0.08) for VGG-16. However, 95% confidence intervals for malignancy predictions are overlapping for each model across sites. It is noteworthy that the unguided prototype model, ProtoPNet, experiences the largest mean AUC drop.

On iCAD’s 99.6% malignant dataset, IAIA-BL issued either overwhelmingly low likelihood of malignancy or high likelihood predictions (<0.2, >0.8), leading to a mean prediction of 0.51 with a large standard deviation of 0.45. VGG16’s behavior was similar but exchanged some low likelihood predictions for medium likelihood predictions, leading to a mean of 0.59 and standard deviation of 0.34. ProtoPNet’s distribution had a lower mean (0.45) and standard deviation (0.25). Given the high incidence of malignancy in the dataset, no model performed exceptionally, but VGG-16 and IAIA-BL both correctly identified a large number of cancers.

## Discussion

### Distributional shifts

It is likely the drops in performance are the result of a change in the data distributions between the institutions since all models experience similar effects. Degradation of model performance when moving between institutions has been observed in many medical imaging settings [21,22]. This problem becomes worse with small sample sizes.

Castro *et al*. propose a taxonomy of five types of distributional shifts between training and testing in medical imaging that may impede model generalization, all of which are relevant to our setting [23]. We inventory these shift types as a case study.

Two of the shift types are common concerns in medical studies: *Population Shift* occurs when the underlying patient populations at the training and test site are different; and, *Prevalence Shift* occurs when the prevalence of the underlying condition changes at the two sites, independent of patient demographics. At Emory, the patient population skewed younger than at Duke, whereas the iCAD dataset skewed older. Emory university has a higher proportion of Black/African American patients than the original training set (Table 1). iCAD does not report patient racial demographics, so it is possible the patient population is significantly different from the other two sites. 27 out of 103 cases were malignant at Emory and 447 out of 449 at iCAD, whereas 36 out of 78 in the Duke test dataset were malignant.

The other three shift types in the Castro taxonomy are specific to machine learning, medical imaging, and their intersection. *Acquisition Shift* is a change in the procedure for data collection. The Duke dataset was collected from 80% GE machines (n=63) and 20% (n = 15) Hologic machines. Emory was collected from 100% Hologic machines (n = 103). iCAD was 50% GE (n = 234), 27% Hologic (n = 124), and 23% Seimens (n = 139). In addition, images were prepared at Emory using a proprietary software to apply window and leveling (prior to this study), whereas images at Duke and iCAD were prepared by applying windowing and leveling to the raw DICOMs.

*Manifestation Shift* occurs when the presentation of the disease varies between sites. The original training set was relatively small for a difficult medical imaging task like tumor classification (n = 516). Given the degradation in performance across all model types, it is likely there are manifestations of both benign and malignant cases that the models were not exposed to during training. This may have been exacerbated by the deliberate choice to classify only three of the five BI-RADS margin types.

Finally, *Annotation Shift* is a difference in the annotation procedure, including differences between annotators. It is likely that annotator disagreement plays a significant role in margin classification performance. While malignancy labels are collected from confirmed histopathology on all datasets, the margin labels are a matter of judgment by a trained radiologist. Margin labels have been found to elicit moderate inter-annotator disagreement [24]. The Duke in-house dataset was annotated by a single fellowship-trained mammography sub-specialist, and therefore the test and train data had ideal inter-annotation agreement. The annotations for the Emory and iCAD datasets were provided by multiple radiologists. Moreover, the purpose of the label collection was different across the sites: At Emory, the labels were collected as part of recording BI-RADS outcomes during screening. At iCAD, like at Duke, the annotations were added specifically for training and testing machine learning models, but annotators only determined if the margin was spiculated. As a result, the results on malignancy prediction performance are more reliable than the results on margin classification performance.

A second form of annotation shift arises from the preparation of the ROIs. Like the margin annotations, ROIs bounding boxes at Duke were prepared by a single radiologist for machine learning training. ROIs at iCAD were created by placing a bounding box around pixel-level outlines of the lesion that were for machine learning training, leading to a tight fit around the lesion. Conversely, the ROI annotations at Emory were collected from radiologists highlighting areas for clinical inspection. Qualitative inspection of the ROIs from Emory University suggested they are looser around the lesion than both iCAD and the original Duke dataset.

Ideally, guiding a model to learn only the decision-relevant portions of the image would improve robustness to all of these shifts. While adding fine annotation training made IAIA-BL more robust to these shifts than unaltered ProtoPNet, it did not improve over blackbox VGG16. As a result, we learned that faithful reasoning restricted to decision-relevant portions of the images is not sufficient to imbue robustness to distribution shifts that occur between different medical image collection sites and conditions.

### Activation robustness

Distributional shifts often cause performance degradation when models learn spurious correlations in their training data, like unrelated features of background [25]. While we noted in the previous section that faithful activation maps concentrated on the lesion are not sufficient to ensure generalization, they are sufficient to avoid background confounding. We know that background confounding is *not* the explanation for IAIA-BL’s failure to generalize, because IAIA-BL maintained its high activation precision at all sites, indicating the model has indeed learned to look at features of the lesion itself and not the surrounding tissue. This is in contrast to ProtoPNet, which experiences a drop in activation precision and larger drop in prediction performance than IAIA-BL, suggesting it has learned some confounding information in training.

These results suggests that the fine annotation used to guide IAIA-BL’s prototypical reasoning can be robust to changes in data distribution, even for a difficult domain like medical imaging. In fact, activation precision actually improved on the Emory dataset, but this may partly be explained by differences in ROI sizes relative to the lesions at the different sites. Larger bounding boxes, like at Emory, are likely to improve activation precision, because there is more area to be counted positively, even if it is not actually from the lesion itself. In this light, differences in ROI sizes provide a plausible explanation for how performance could decrease on the Emory dataset while activation precision increased. Moreover, it can explain why activation precision on iCAD was lower for all models than on the Duke dataset.

The generalization of activation precision is noteworthy because the implementation of fine annotation loss is lightweight—only 15 out of 516 training images had fine annotation labels—and applicable to other methods. The fine annotation loss term penalizes the model for “activating” highly outside the annotated region during training. “Activation” in the context of IAIA-BL is similarity between the samples and learned prototypical lesions. Beyond IAIA-BL, there is a large literature on generating activation maps using other methods (for instance, we use GradCAM and GradCAM++ in this work for comparison). Fine annotation loss is a paradigm for guiding model reasoning that can be applied to any method that produces an activation map. However, we caution that such implementations may lead to false confidence if applied to post-hoc activation maps. While IAIA-BL’s activation maps are faithful representations of its reasoning, this is not true of standard post-hoc methods like GradCAM. Using fine annotation with post-hoc methods may lead to performance improvements and more concentrated activation maps irrespective of their faithfulness. Instead, researchers should leverage fine annotations to improve performance while employing faithful activation maps, like those of IAIA-BL.

### Limitations

Along with the limitations inherent to any retrospective study, this study has three notable limitations. First, IAIA-BL was trained on only three of the five BI-RADS mass margins. As a result, the data selected from iCAD (which only had labels of spiculated and non-spiculated) may include margins that IAIA-BL is not trained to differentiate, which we expect to negatively impact malignancy and mass margin prediction performance. Moreover, the iCAD dataset was extremely unbalanced (99.6% malignant), making AUC a poor metric for performance. Second, the five-part criteria for case inclusion reduced the number of cases available to this study to a small fraction of the total datasets, particularly for EMBED. Third, there is known inter-annotator disagreement when labeling mass margins, and labels from each site were acquired with different views and metadata available to annotators at that site. Those labels were used for both data selection and measurement of mass margin prediction performance. It is possible that many of these limitations could be mitigated if IAIA-BL were trained on a larger and more complete dataset.

## Conclusion

A retrospective external validation of the IAIA-BL breast lesion malignancy prediction model from Barnett *et al*. [4] was completed at two sites, Emory University (56 patients) and iCAD (303 patients). Malignancy prediction performance was lower at Emory University as measured by AUC, but this drop in performance also occurred for the baseline models (ProtoPNet, VGG16), and the confidence intervals for all models at each site overlapped. All of the models displayed a wide distribution of malignancy scores on the iCAD dataset which included almost no benign lesions (99.6% malignant lesions).

Margin classification, which is instrumental to the IAIA-BL malignancy predictions and self-explanations, showed clear degradation as measured by AUC at all sites for all models. While this is likely, in part, due to differences in data distribution, labeling differences may also play a key role. The Duke University data was all labeled by the same radiologist, whereas data from external sites was labeled by multiple radiologists.

Activation precision was used to quantify the model’s exclusive focus on the lesion. The activation precision was consistently high for IAIA-BL but not for baseline ProtoPNet or its black box counterpart VGG-16 with GradCAM++. This suggests that the fine annotation training defined for this task by Barnett *et al*. [4] can teach deep learning models with faithful activation maps to identify clinically relevant features of radiological images, providing a mechanism to limit reliance on background confounders.

We reach two conclusions. First, the specific model would need to show better generalization to ensure its malignancy prediction maintains performance acceptable for clinical use; all of the models tested shown substantial decreases in predictive performance for the tasks of mass margin and malignancy prediction. Second, the interpretability of IAIA-BL was maintained across all sites. This provides strong evidence in favor of using a fine annotation loss to provide spatial guidance to faithful activation maps for deep learning models, and a proof-of-concept of doing so using a prototypical-part model for medical imaging. While this is not sufficient to ensure performance generalization, it can prevent background confounding, as in our study, and will hopefully enable better troubleshooting by domain experts.

## Supporting information

S1 Appendix Context window tuning and EMBED Open Data results (PDF)

S1 Tables EMBED Open Data case demographics. (PDF)

S1 Fig Margin classification ROC curves. *A. EMBED Open Data*. *B. Emory EMBED.* ROC curves are provided for IAIA-BL, ProtoPNet, and the black box baseline VGG16. AUCs are reported in the legend, and 95% confidence intervals, calculated using DeLong’s method, are in parentheses. Confidence intervals are omitted for the single spiculated margin.
(TIFF)

S2 Fig EMBED open data margin level activation precision. Activation Precision of margin prediction for IAIA-BL and baseline models. VGG-16 does not have a self-explanation of activation, so GradCAM and GradCAM++ were used. Error bars represent 95% confidence intervals, calculated using bootstrap resampling (n = 100). IAIA-BL had perfect activation precision for all margin classes in EMBED Open Data (1.00).
(TIFF)

S3 Fig EMBED open data malignancy prediction ROC curves. AUCs are reported in the legend. 95% confidence intervals, calculated using DeLong’s method, are in parentheses. VGG16 had perfect malignancy prediction on the EMBED Open Data subset so its confidence intervals are omitted.
(TIFF)

S1 Data Margin classification and malignancy prediction model evaluation results. This dataset contains model evaluation metrics (ROC curves, confidence intervals, activation maps), demographic information from four medical imaging sites (Duke, Emory, iCAD, and EMBED Open Data) used in the study, and logits for replication of EMBED Open Data results.
(ZIP)

S1 Source Code Source Code for training and evaluating IAIA-BL at study sites. Based off of https://github.com/alinajadebarnett/iaiabl.
(ZIP)
