# Supplementary material for: Multi-site validation of an interpretable model to analyze breast masses
Source: PLoS One. 2025 Jun 26;20(6):e0320091. doi: 10.1371/journal.pone.0320091 (PMC12200715; doi:10.1371/journal.pone.0320091)
Supplement: S1 Appendix — (PDF) [file pone.0320091.s001.pdf]

S1 Appendix

Context Window Size Tuning.

Context windows sizes are expressed in pixels from a single side of a bounding box. For instance, for a 100x100 square pixel ROI, and context window of size 50 pixels yields a new image of 200x200 pixels. The original ROI is centered in the new image, such that the edges of the original ROI are 50 pixels away from each edge of the image. Likewise, a negative context window size *removes* pixels from the ROI as measured from the ROI edge.

Context window size was tuned on Emory EMBED on a validation set of 100 randomly sampled patients with confirmed masses and histopathologies, but without margin labels. For each patient, a grid search of context window sizes was conducted over the following context window sizes (in pixels): -100, -75, -50, -25, 0, 25, 50, 75, 100.

Malignancy prediction was most performant at a context window size of 100, as measured by AUC. This is the same size used in the original IAIA-BL paper. Therefore, a context window size of 100 was used in the final analysis.

EMBED Open Data Results.

Results using the open access subset of EMBED, *EMBED Open Data*, are reported here split-out from the full EMBED dataset. EMBED Open Data contains cohorts 1 and 2 of EMBED, which has 12 cohorts in total. EMBED Open Data is available at `https://registry.opendata.aws/emory-breast-imaging-dataset-embed/`.

Along with results reported in this appendix, sample-level predictions for both malignancy and margins are available for the EMBED Open Data subset in S1 Data, `embed-open-data_logits.csv`.

Data Preparation. The data selection process for EMBED Open Data was identical to EMBED as reported in the Materials and Methods section. Image preparation for EMBED Open Data was also the same as EMBED with one exception: images were extracted from DICOMs using the `ImageMagick` library (v6.9.11-60) invoked using the `OncoData` library (d6c34dd)[1]. Windowing and leveling were applied by ImageMagick.

Demographics. After applying the five-part selection criteria, there were 19 images of 19 lesions from 10 patients in the selected EMBED Open Data dataset. Nine of the masses had circumscribed margins, nine had indistinct margins, and one had a spiculated margin (S1 Tables). Patient demographics are similar to those of EMBED overall, except that all images are from Hologic, Inc. devices (S1 Tables).

Results. All analysis is identical to that reported in Materials and Methods, except that 1) bootstraps that did not include individual classes were resampled to ensure metrics were calculable, and 2) malignancy distributions were omitted since there are both benign and malignant samples. Because of the small sample size (19 samples), confidence intervals are generally wide.

Margin prediction AUC on EMBED Open Data is similar to EMBED with indistinct margins being particularly difficult (S1 Figure). For instance, IAIA-BL has a 0.41 (95% CI: 0.13-0.69) indistinct margin classification AUC on EMBED Open Data and a 0.50 (95% CI: 0.39-0.62) on EMBED, and VGG-19 has 0.39 (95% CI: 0.12-0.66) and 0.45 (95% CI: 0.34-0.56) on the same. Because there was only one spiculated margin, there are no confidence intervals for spiculated margin AUC, but the AUC was .78, .89, and .89 for IAIA-BL, ProtoPNet, and VGG-16, respectively.

Activation precision was likewise similar in means. Noteably, IAIA-BL had perfect activation precision (1.00) on EMBED Open Data (S2 Figure).

[1] `https://github.com/yala/OncoData_Public`

Finally, mean malignancy prediction for VGG-16 and IAIA-BL are higher on EMBED Open Data than EMBED (1.00 and .97, respectively) (S3 Figure). This may be because EMBED Open Data's labeled margins match well with their malignancy. All circumscribed lesions are benign, the spiculated lesion is malignant, and four-of-nine lesions with indistinct margins are malignant. Because VGG-16 classified malignancy perfectly on this subset, it does not have confidence intervals.
