# Supplementary material for: Multi-site validation of an interpretable model to analyze breast masses
Source: PLoS One. 2025 Jun 26;20(6):e0320091. doi: 10.1371/journal.pone.0320091 (PMC12200715; doi:10.1371/journal.pone.0320091)
Supplement: S1 Tables — (PDF) [file pone.0320091.s002.pdf]

S1. Tables

Table 1. EMBED Open Data Patient Test Set Mass Margins

	EMBED Open Data	EMBED (Emory)
Circumscribed	9	47
Indistinct	9	39
Spiculated	1	17

Distribution of mass margins in each test dataset.

Table 2. EMBED and EMBED Open Data Test Set Demographics

	EMBED Open Data	EMBED (Emory)
Unique Patients	10	58
Unique Masses	19	103
Unique Images	19	103
Manufacturer		
Hologic, Inc.	19	103
Patient Age		
< 50	7	31
50-69	7	47
≥ 70	5	25
Race		
Black or African American	7	49
Caucasian/White	12	47
Other/Unknown	-	7

All values represent the number of images except those in the Unique Patients and Unique Masses. "Other" race category includes `Asian`, `Native Hawaiian or Other Pacific Islander`, `American Indian or Alaskan Native`, and `Unknown`. Race demographics were not available for the iCAD data.
