# Supplementary figures and images for: Multi-site validation of an interpretable model to analyze breast masses
Source: PLoS One. 2025 Jun 26;20(6):e0320091. doi: 10.1371/journal.pone.0320091 (PMC12200715; doi:10.1371/journal.pone.0320091)

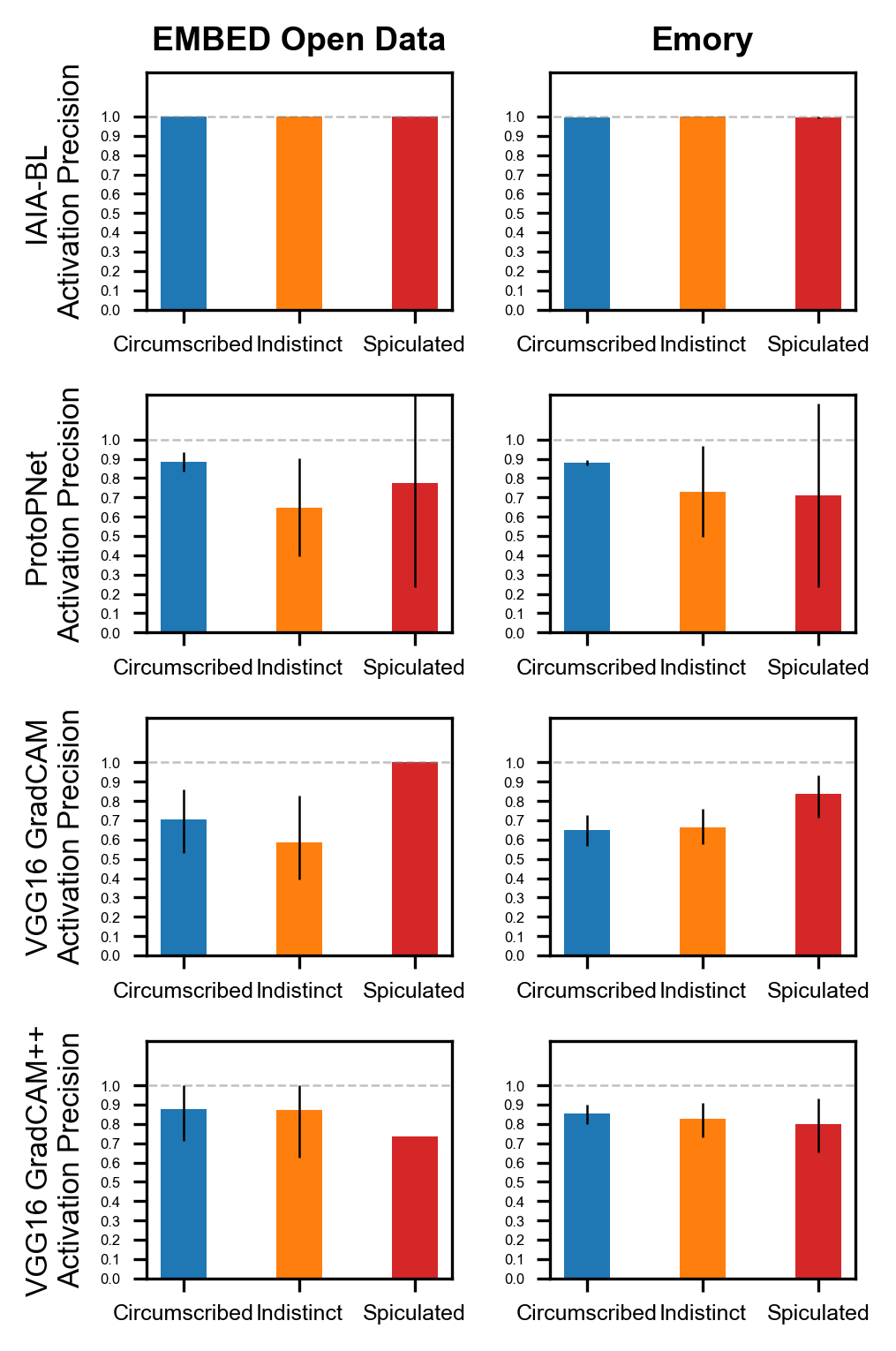

Supplement: S1 Fig — A. EMBED Open Data. B. Emory EMBED. ROC curves are provided for IAIA-BL, ProtoPNet, and the black box baseline VGG16. AUCs are reported in the legend, and 95% confidence intervals, calculated using DeLong’s method, are in parentheses. Confidence intervals are omitted for the single spiculated margin. (TIFF) [file pone.0320091.s003.tif]

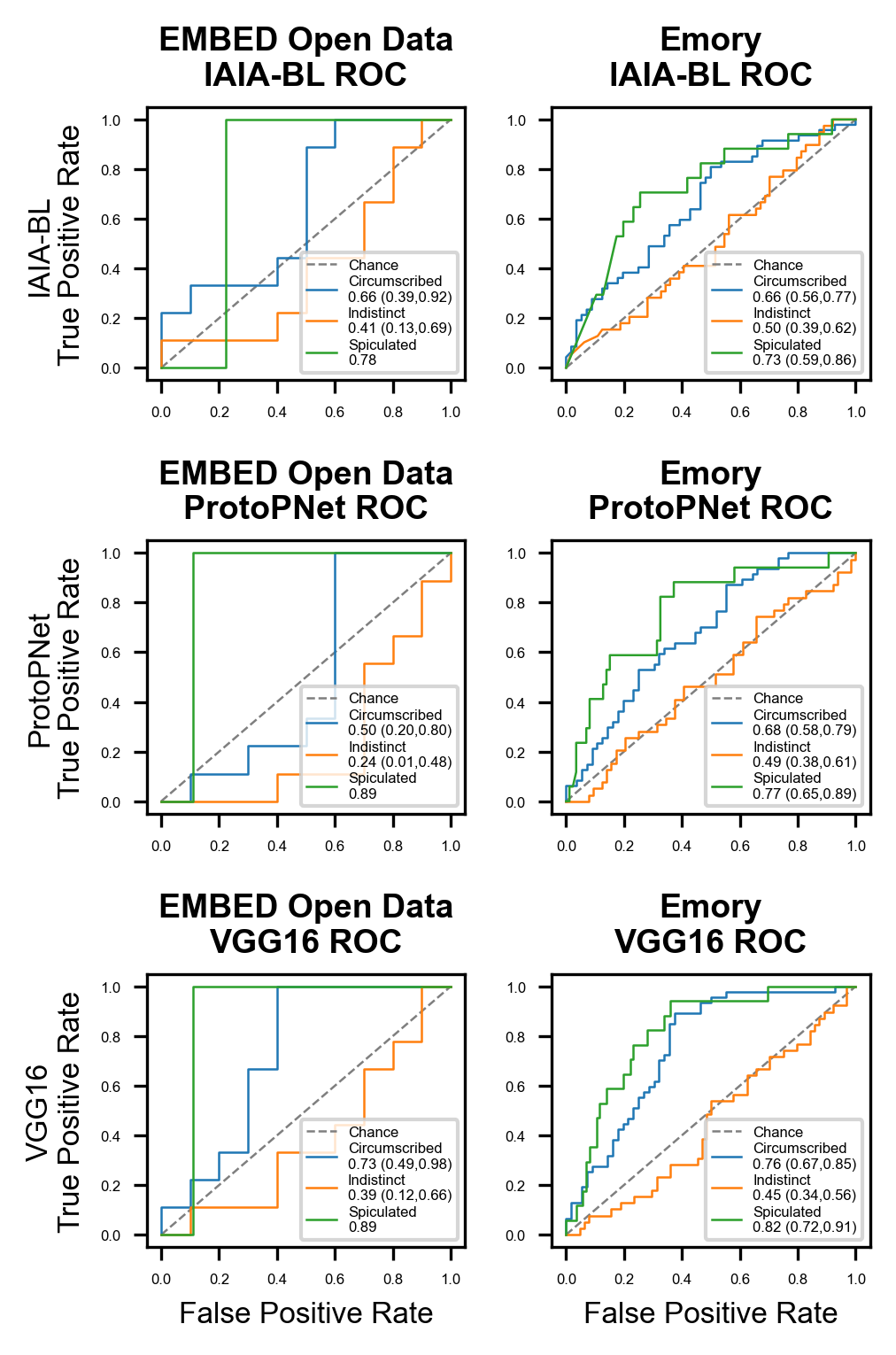

Supplement: S2 Fig — Activation Precision of margin prediction for IAIA-BL and baseline models. VGG-16 does not have a self-explanation of activation, so GradCAM and GradCAM++ were used. Error bars represent 95% confidence intervals, calculated using bootstrap resampling (n = 100). IAIA-BL had perfect activation precision for all margin classes in EMBED Open Data (1.00). (TIFF) [file pone.0320091.s004.tif]

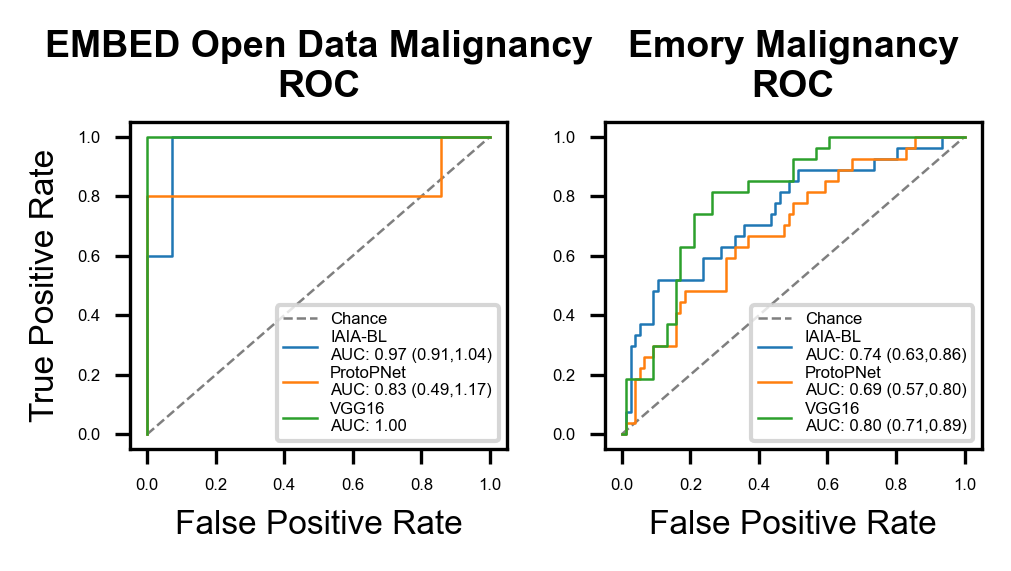

Supplement: S3 Fig — AUCs are reported in the legend. 95% confidence intervals, calculated using DeLong’s method, are in parentheses. VGG16 had perfect malignancy prediction on the EMBED Open Data subset so its confidence intervals are omitted. (TIFF) [file pone.0320091.s005.tif]
